# Can cognitive assessment really discriminate early stages of Alzheimer’s and behavioural variant frontotemporal dementia at initial clinical presentation?

**DOI:** 10.1186/s13195-017-0287-1

**Published:** 2017-08-09

**Authors:** Sophia Reul, Hubertus Lohmann, Heinz Wiendl, Thomas Duning, Andreas Johnen

**Affiliations:** Department of Neurology, University Hospital Münster, Albert-Schweitzer-Campus 1, 48149 Münster, Germany

**Keywords:** Alzheimer’s dementia (AD), Behavioural variant frontotemporal dementia (bvFTD), Differential diagnosis, Neuropsychological tests

## Abstract

**Background:**

Neuropsychological testing is considered crucial for differential diagnosis of Alzheimer’s disease (AD) and behavioural variant frontotemporal dementia (bvFTD). In-depth neuropsychological assessment revealed specific dysfunctions in the two dementia syndromes. However, a significant overlap of cognitive impairments exists in early disease stages. We questioned whether a standard neuropsychological assessment at initial clinical presentation can delineate patients with AD versus bvFTD.

**Methods:**

In a retrospective approach, we evaluated and compared how cognitive profiles assessed at initial clinical presentation predicted the diagnosis of later verified AD (*n* = 43) and bvFTD (*n* = 26). Additionally, the neuropsychological standard domains memory, language, visuospatial skills, executive functions, praxis and social cognition were subjected to stepwise discriminant analysis to compare their differential contribution to diagnosis.

**Results:**

Regardless of diagnosis, a percentage of patients presented with major deterioration in a wide range of cognitive domains when compared with age-matched normative data. Only few significant differences were detected on the group level: Patients with AD were relatively more impaired in the *verbal recall*, *verbal recognition*, *figure copy*, and surprisingly in the executive subdomains, *set shifting* and *processing speed* whereas bvFTD was characterised by more deficits in *imitation of face postures*. A combination of tests for *verbal recall*, *imitation of limb and face postures*, and *figure copy* showed the greatest discriminatory power.

**Conclusions:**

Our results imply that the contribution of a standard neuropsychological assessment is limited for differential diagnosis of AD and bvFTD at initial presentation. In contrast to current clinical guidelines, executive functions are neither particularly nor exclusively impaired in patients with bvFTD when assessed within a standard clinical neuropsychological test battery. The significant overlap of bvFTD and AD concerning the profile of cognitive impairments questions current neuropsychological diagnostic criteria and calls for revision, regarding both the degree and the profile of cognitive deficits.

## Background

Behavioural variant frontotemporal dementia (bvFTD) and Alzheimer’s disease (AD) are two of the most common dementia syndromes affecting people under the age of 65 [1]. A correct and early differential diagnosis is crucial for disease management and treatment [2]. In patients with suspected dementia, a comprehensive neuropsychological examination is an essential diagnostic element besides history taking, evaluation of regional brain atrophy on magnetic resonance imaging (MRI), and a cerebrospinal fluid (CSF) biomarker profile. Clinically, AD is characterised by progressive cognitive decline, especially in episodic memory, spatial perception and working memory, deriving from temporal and medial parietal lobe atrophy [3]. In contrast, patients with bvFTD predominantly present with severe behavioural deterioration, personality changes and social dysfunction, associated with prominent frontal and anterior temporal lobe atrophy [4]. Besides the behavioural conspicuities, a typical cognitive profile described as “executive deficits with relative sparing of memory and visuospatial functions” (page 2460) represents one of the six diagnostic criteria [4]. The neuropsychological item has been described as highly sensitive for bvFTD [5]. Therefore, testing for cognitive dysfunction is an important and auxiliary element in the diagnostic process. However, recent evidence has revealed that patients with bvFTD also show a range of cognitive deficits similar to those found in patients with AD, especially in early stages of the disease [6, 7]. In particular, standard memory tests have failed to reliably discriminate the two diseases [8]. Due to such clinical overlap, the differentiation of bvFTD and AD in early disease stages remains challenging [6, 9, 10]. To address the issue of partly overlapping cognitive impairments in AD and bvFTD, researchers in several recent studies did in-depth investigations of neuropsychological domains such as memory, executive function, praxis and social cognition [8, 11–21]. These studies succeeded in delineating the two diseases by investigating single cognitive domains with extensive but often time-consuming neuropsychological test batteries. However, it remains unclear whether a standard neuropsychological examination as usually applied in specialised memory clinics actually contributes to correct differentiation between AD and bvFTD and which of the aforementioned neuropsychological domains has the highest value for differential diagnosis.

To investigate this, we retrospectively examined cognitive profiles of patients with suspected AD and bvFTD assessed at initial clinical presentation. Importantly, all diagnoses were later supported by brain imaging results, biomarker evidence of the underlying pathological process and typical disease progression documented by clinical follow-up presentations. We analysed which cognitive domains provided the most efficient differentiation between the dementia groups and which did not yield incremental information for the differential diagnosis and may thus be neglected in a standard neuropsychological test battery for the differentiation of AD and bvFTD.

## Methods

### Participants

**Fig. 1:**
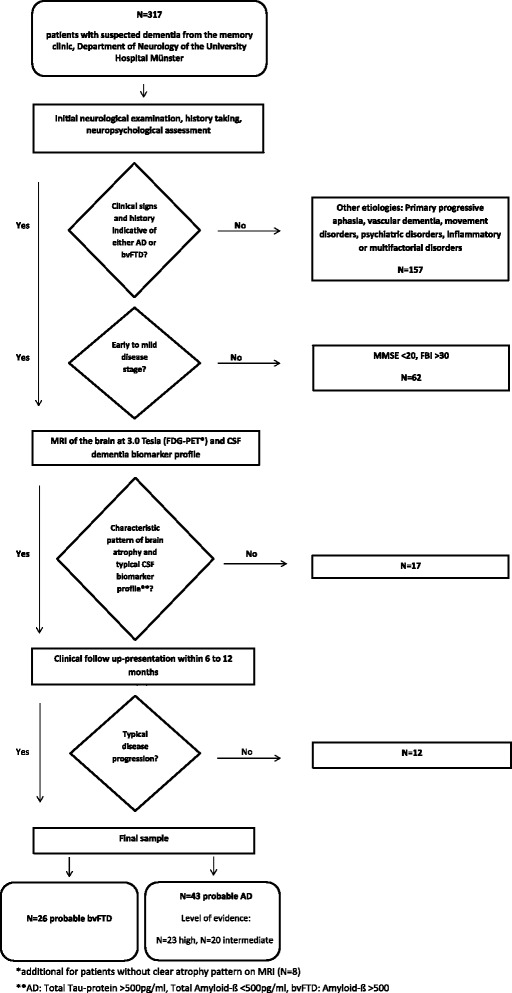
Flowchart illustrating participant selection and exclusion criteria

Figure 1 depicts the participant selection and exclusion process for the present study. A total of 317 participants were recruited from the memory clinic at the Department of Neurology of the University Hospital Münster, Germany, between August 2011 and September 2014, where they presented with signs of dementia. For initial diagnostic evaluation, all participants routinely underwent a neurological examination and a detailed neuropsychological anamnesis and assessment. Initially, 157 patients with signs of aetiologies other than bvFTD or AD, including clinically predominant aphasia, major vascular impairment, movement disorders, depression, psychiatric disorders, and inflammatory or multifactorial aetiologies were excluded. Subsequently, patients with advanced disease progression were excluded from the present study; disease duration measured as time since first notice of symptoms was documented by caregivers and in clinical records of all patients. For patients with suspected AD, mild disease stages were moreover assumed only when presenting with a Mini Mental State Examination (MMSE) score >20 [22, 23]. In case of suspected bvFTD, disease progression was additionally estimated with the Frontal Behavioral Inventory (FBI), a caregiver report used to determine severity of behavioural changes in bvFTD [24, 25]. Early and mild stages were assumed for scores between 25 and 30 points in accordance with the test manual [24]. Using these rigid criteria for disease severity, we excluded 62 patients with advanced disease progression (MMSE <20 points and/or FBI >30 points). For the remaining patients, results of MRI of the brain at 3.0 Tesla and CSF biomarker profiles were retrospectively checked to confirm the diagnosis. ^18^F-fluorodeoxyglucose positron emission tomography (FDG-PET) was additionally applied in a subsample of patients with bvFTD (*n* = 8) when no local atrophy pattern was visible on MRI studies. FDG-PET revealed a typical bifrontal hypometabolism. Particularly, we aimed to eliminate the possibility of an underlying AD pathology in patients with suspected bvFTD. We excluded 17 patients with a less-marked brain atrophy pattern and/or with a conflicting CSF biomarker profile for the respective diagnosis (i.e., patients with suspected AD but without the typical constellation of decreased amyloid-β [<500 pg/ml] and increased tau protein [>500 pg/ml], as well as patients with suspected bvFTD showing pathologically decreased amyloid-β [<500 pg/ml]). To further support the suspected diagnoses, all included patients had a clinical follow-up presentation in our memory clinic within 6–12 months after their initial presentation. We excluded another 12 patients on the basis of non-typical clinical disease progression. Finally, we classified the remaining patients in accordance with current diagnostic criteria into different levels of diagnostic certainty [3, 4]. The study was approved by the local ethics committee (2012-365-f-S). All participants gave written informed consent.

### Neuropsychological assessment

Neuropsychological tests measure complex domains of human cognition such as attention, perception, memory, language, executive function and emotional processing with quantitative and standardised methods [26]. Table 1 summarises the employed neuropsychological tests with their corresponding cognitive domains and subdomains. Well-established and standardised psychometric tests covering the cognitive domains *memory*, *language*, *visuospatial skills*, *attention*, *executive function*, *praxis* and *social cognition* were applied (Table 1). To compare patients with appropriate age-matched normative data and to prevent floor or ceiling effects, two different test programmes for younger and older patients were used to cover these domains (Table 1, italicised tests for patients aged >65 years). All tests were carried out by experienced clinical neuropsychologists (SR, HL, AJ).

**Table 1:** Standard neuropsychological assessments used in this study

Cognitive domain and subdomain^a^	Cognitive tests	First author, publication date [reference]
Dementia screening	MMSE	Folstein, 1975 [22]
Memory		
Verbal span^a^	VLMT trial 1	Helmstaedter, 2001 [46]
	*CERAD wordlist trial 1*	Aebi, 2002 [47]
Verbal learning^a^	VLMT trial 1–5	see above
	*CERAD wordlist trial 1–3*	see above
Verbal recall^a^	VLMT trial 6	see above
	*CERAD wordlist recall*	see above
Verbal recognition^a^	VLMT trial 8 true	see above
	*CERAD wordlist recognition %*	see above
Visual recall^a^	CFT 3-minute recall	Meyers, 1996 [48]
	*CERAD figure recall*	see above
Language		
Object naming^a^	Wortproduktionsprüfung (Word Production Test)	Blanken, 1999 [49]
	*CERAD Boston naming test 15*	see above
Visuospatial skills		
Figure copy^a^	CFT copy	see above
	*CERAD figure copy*	see above
Executive function		
Semantic word fluency ^a^	RWT- 1 minute category fluency “animals”	Aschenbrenner, 2000 [50]
Phonematic word fluency ^a^	RWT- 1 minute letter fluency “S”	see above
Set shifting^a^	TMT B	Tombaugh, 2004 [51]
Digit span backwards^a^	Wechsler Memory Scale – backward digit span	Härting, 2000 [52]
Attention		
Processing speed^a^	TMT A	see above
Praxis		
Pantomime of object use^a^	Cologne Apraxia Screening 1.1 and 1.2.	Weiss, 2013 [53]
Imitation of limb postures^a^	Cologne Apraxia Screening 2.2	see above
Imitation of face postures^a^	Cologne Apraxia Screening 2.1	see above
Social cognition		
Facial emotion recognition^a^	Ekman Facial Emotion Recognition test (SEA)	Funkiewiez, 2012 [54]

*Abbreviations*: *CERAD* Consortium to Establish a Registry for Alzheimer’s Disease neuropsychological test battery, *CFT* Complex Figure Test, *MMSE* Mini Mental State Examination, *RWT* Regensburg Word Fluency Test, *SEA* Social Cognition and Emotional Assessment, *TMT* Trail Making Test, *VLMT* Verbal Learning and Memory Test

Column 1 displays all covered cognitive domains (*bold*) and subdomains. Column 2 displays the incorporated tests to cover the respective subdomains. Italicised tests display the alternative test set, which was employed for patients >65 years of age (Alzheimer’s disease 18 of 43, behavioural variant of frontotemporal dementia 4 of 26). For inter-test and inter-subdomain comparability in statistical analysis, individual test raw scores were z-transformed. Column 3 presents the first author’s name and the publication date of the normative data we used to estimate patients’ test performance

^a^Used for statistical analysis

### Statistical analyses

Statistical analyses were performed using IBM SPSS Statistics version 22 software (IBM, Armonk, NY, USA). Cognitive impairment of the patients in each subdomain was evaluated by comparing their test performance with age-matched normative sample data available from the particular test’s manual. Individual test scores lower than −1.5 SD from the standardised mean were classified as below average and thus to indicate cognitive impairment [26]. Between the patient groups, demographic data and cognitive test results were compared via *t* tests. For inter-test and inter-subdomain comparability, raw test scores were transformed into z-scores so that we were able to compare patients’ performance in the different cognitive subdomains without regard to the employed test procedures. Prior to analysis, variables were plotted, and normality of distribution was confirmed by the Kolmogorov-Smirnov test. To correct for multiple comparisons, the significance level for group differences in cognitive performance was adjusted for the number of cognitive tests using the Bonferroni-Holm correction (*p* < 0.003). To determine how well patients with AD and patients with bvFTD can be distinguished on the basis of neuropsychological test battery and to assess the differential contribution of the subdomains, a stepwise discriminant function analysis was performed. As predictors, we included all cognitive variables that fulfilled significant group differentiation between AD and bvFTD at a significance level of α < 0.05. On the basis of this analysis, all discriminating variables can be ranked for their importance for successful group discrimination by standardised canonical discriminant function coefficients, in which higher values indicate a greater impact on the final group classification. Before discriminant function analysis, the variance-covariance matrix was checked for strong inhomogeneity. Subsequently, a jack-knifed classification procedure was computed to validate the statistical prognosis of the computed model. This special case of resampling method classifies each case on the basis of functions derived from all other cases and on the basis of prior probabilities computed from sample size. Multiple imputation methods were employed to maintain statistical reliability in case of missing data.

## Results

### Level of diagnostic evidence of the final sample

All patients who passed the diagnostic process were subsequently classified in accordance with current diagnostic guidelines [3, 4] regarding disease-specific pathophysiological changes (Fig. 1). Structural MRI of the brain at 3.0 Tesla was available for all participants. Additionally, analysis of CSF biomarker profiles was available for 38 patients with AD and 25 patients with bvFTD. All 43 patients with suspected AD met criteria for probable AD. Of these, 23 patients fulfilled high-level evidence and 20 had an intermediate level of evidence for disease-specific pathophysiological changes according to the McKhann et al. criteria [3]. All 26 patients with suspected bvFTD fulfilled the criteria for probable bvFTD. Comparison of CSF biomarker profiles between the two patient groups revealed significantly lower total amyloid-β peptide levels for the AD group, whereas total tau protein was significantly lower in the bvFTD group (Table 2).

**Table 2:** Demographic, clinical and cognitive data of patients with behavioural variant of frontotemporal dementia and patients with Alzheimer’s disease at initial clinical presentation

	bvFTD (*n* = 26)	AD (*n* = 43)	bvFTD vs. AD	Classification as bvFTD vs. AD
	Mean (SD)	Mean (SD)	*t*(*df*)	
Demographics				
Age, years	65 (8)	72 (9)	3.44 (67)^a^	
Education, years	11 (1.7)	11 (1.8)	−0.98 (67)	
Sex, M/F	21/5	17/26	N/A	
Disease severity scores				
MMSE	26 (3)	24 (3)	−2.48 (67)^b^	
FBI	28 (10)	N/A	N/A	
Disease duration, months	29 (19)	23 (14)	−1.40 (67)	
Total amyloid-β	850 (424)	397 (108)	−4.16 (61)^a^	
Total tau protein	403 (256)	756 (277)	4.07 (61)^a^	
Neuropsychological assessment				
Memory				
Verbal span	−0.77 (0.98)	−1.47 (0.99)	−2.88 (67)^b^	
Verbal learning	−1.54 (1.32)	−2.36 (1.11)	−2.77 (67)^b^	
Verbal recall	−1.74 (1.25)	−2.98 (0.77)	−5.1 (67)^a^	*0.613*
Verbal recognition	−0.77 (1.74)	−4.14 (5.25)	−3.88 (67)^a^	
Visual recall	−0.74 (0.95)	−1.34 (0.90)	−2.62 (67)^b^	
Language				
Object naming	−0.16 (0.94)	−1.06 (1.55)	−2.99 (67)^b^	
Visuospatial skills				
Figure copy	0.16 (1.05)	−1.05 (1.85)	−3.48 (67)^a^	*0.300*
Executive functions				
Semantic word fluency	−1.11 (0.98)	−1.21 (1.05)	−0.43 (67)	
Phonematic word fluency	−1.13 (0.88)	−0.58 (1.14)	2.08 (67)	
Set shifting	−1.50 (1.90)	−3.18 (2.15)	−3.18 (67)^a^	
Digit span backwards	−1.29 (1.39)	−1.71 (1.23)	−1.26 (67)	
Attention				
Processing speed	−0.90 (2.31)	−3.48 (3.73)	−3.41 (67)^a^	
Praxis				
Pantomime of object use	−2.51 (2.04)	−5.21 (5.90)	−2.17 (67)^b^	
Imitation of limb postures	−2.80 (3.41)	−5.39 (4.61)	−2.45 (67)^b^	*0.299*
Imitation of face postures	−4.88 (4.04)	−2.00 (2.50)	3.33 (67)^a^	*−0.512*
Social cognition				
Facial emotion recognition	−2.35 (2.41)	−1.96 (2.10)	0.58 (67)	

*Abbreviations: AD* Alzheimer’s disease, *bvFTD* Behavioural variant of frontotemporal dementia, *FBI* Frontal Behavioral Inventory, *MMSE* Mini Mental State Examination, *N/A* Not applicable

Column 1 displays all demographic and clinical categories, cognitive domains and respective standardised subdomains. Columns 2 and 3 display group means and SDs of demographic and clinical categories and of the z-transformed cognitive subdomain scores (*refer* to Table 1). Column 4 displays the test statistical values resulting from group comparison analysis (Student’s *t* test). Column 5 displays the *standardised canonical discriminant function coefficients* resulting from discriminant function analysis. Higher values indicate major importance for successful group classification

^a^Significant difference at *p* < 0.003 (Bonferroni correction for number of cognitive tests)

^b^Significant difference at *p* < 0.05

### Demographic data and disease severity scores

Table 2 summarises demographic characteristics of the final sample. Patients with AD were significantly older than patients with bvFTD (bvFTD age 65, AD age 72, *p* < 0.001). Patient groups did not differ regarding years of education. There was a significantly higher percentage of male patients in the bvFTD group than in the AD group (χ^2^ = 11.13, *df* = 1, *p* = 0.001). Disease duration was comparable in both patient groups (bvFTD 29 months, AD 23 months, *p* = 0.199). Patients with AD had significantly lower MMSE scores than patients with bvFTD (bvFTD 26, AD 24, *p* = 0.018). The average FBI score for all patients with bvFTD was 28, indicating mild disease status.

### Cognitive test results

#### Individual performance of AD and bvFTD compared with age-matched normative data

**Fig. 2:**
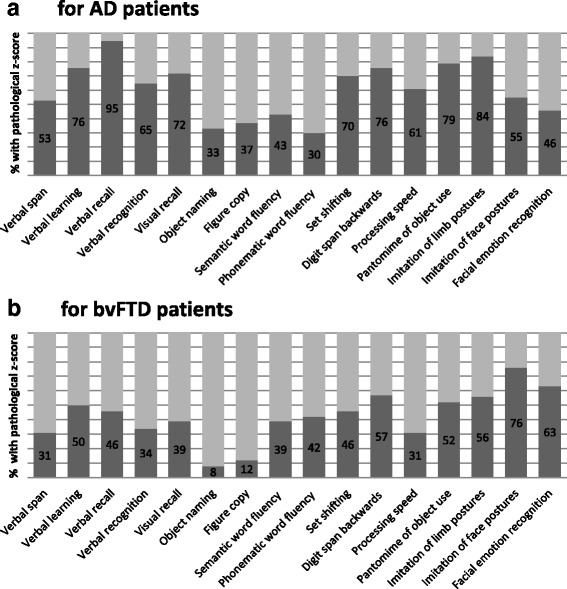
Individual test performance of patients with dementia. Percentage of patients with Alzheimer’s disease (AD) (**a**) and patients with behavioural variant of frontotemporal dementia (bvFTD) (**b**) with pathological z-scores (less than −1.5 SD) adjusted for sex, age and years of education

We first examined the number of impaired cases within AD and bvFTD groups on an individual level. Percentages of patients with impaired performance (<1.5 SD compared with age-matched normative sample data) are presented in Fig. 2 for each patient group separately.

The majority of patients with AD (53–95%) and one-third to one-half of the patients with bvFTD (31–50%) showed significant impairments in verbal and visual memory domains. *Figure copy* performance was disturbed in 37% of those with AD and in only 12% of patients with bvFTD.

Within the executive function domain, the percentage of impaired patients was highly varied, depending on the subdomain. The majority of both patient groups was impaired in the subdomain *digit span backwards* (57% bvFTD, 76% AD), whereas for the subdomain *phonematic* and *semantic word fluency*, about one-third of both dementia groups (30–43%) was impaired. For the subdomains *set shifting* and *processing speed*, the majority of the patients with AD (61–70%) was impaired, whereas less than half of the patients with bvFTD (31–46%) had disturbances.

Within the praxis domain, major impairment was found for both patients groups (55–84% AD, 52–76% bvFTD). For the social cognition domain, 63% of the patients with bvFTD and 46% of the patients with AD revealed impaired *facial emotion recognition* abilities. The least impairment was found for both patients groups in the *object naming* subdomain (8% bvFTD, 33% AD).

#### Direct comparison of group means in AD vs. bvFTD

**Fig. 3:**
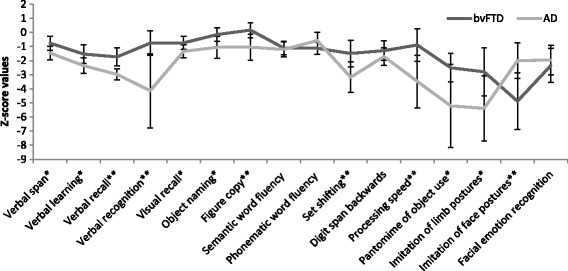
Comparison of cognitive profiles of patients with behavioural variant of frontotemporal dementia (bvFTD) and patients with Alzheimer’s disease (AD). *X*-axis displays cognitive subdomains, and *y*-axis displays z-score values. The *lines* display mean scores for each group in each subdomain. * Significant differences between the groups at a significance level of *p* < 0.05 for the marked subdomain; ** significant differences between the groups at *p* < 0.003 (significance level after Bonferroni correction for number of cognitive tests) for the marked subdomain

We next compared group means of patients with bvFTD and AD directly. The results of between-group comparisons of all cognitive subdomains and results of group classification are presented in Table 2. After Bonferroni-Holm correction for the number of cognitive subdomains (α = 0.003), patients with AD were significantly more impaired than patients with bvFTD in the subdomains *verbal recall* (*p* < 0.001), *verbal recognition* (*p* = 0.002), *figure copy* (*p* = 0.001), *processing speed* (*p* = 0.001) and *set shifting* (*p* = 0.002). Patients with bvFTD showed significantly lower performance in *imitation of face postures* (*p* = 0.001) than patients with AD. These comparisons remained significant after correction for age, sex, education, disease duration and MMSE score as potentially confounding covariates (*see* Table 3). Using a more liberal significance level of α = 0.05, Student’s *t* tests further showed significant differences between AD and bvFTD in performance on *verbal span*, *verbal learning*, *visual recall*, *object naming, pantomime of object use* and *limb imitation*, with slightly poorer performance of patients with AD*.* All other subdomains (*semantic fluency*, *phonematic fluency*, *digit span backwards*, *facial emotion recognition*) showed no statistical group differences between AD and bvFTD. Figure 3 presents and compares cognitive test profiles of the patient groups.

**Table 3:** Results of covariance analysis

	Wilks’ λ		
Effect	Value	*F*	Significance
Age	0.084	2.712	0.173
Sex	0.170	1.220	0.469
Education	0.136	1.591	0.351
MMSE score	0.277	0.653	0.760
Disease duration	0.508	0.242	0.983

*MMSE* Mini Mental State Examination

The table shows statistical values for sex, age, education, MMSE score and disease duration used as covariates in a multivariate analysis of variance including all cognitive subdomains as dependent variables and diagnosis as a fixed factor

#### Classification of AD and bvFTD

The subdomains *verbal recall*, *figure copy*, *imitation of face postures* and *imitation of limb gestures* were statistically selected for the optimal discriminant function in a stepwise process (*refer* to Table 2 for standardised canonical discriminant function coefficients). The resulting discriminant function was significant (Wilks’ λ = 0.484, χ^2^ = 24.305, *p* = 0.028). On the basis of absolute differences within this subdomain combination, a successful classification of patients as bvFTD (in 77%) or AD (in 90%) was possible. In the attention, executive function and social cognition domains, no subdomain showed enough discriminative quality to become part of the discriminant function.

## Discussion

Using an innovative approach, we examined the usefulness and predictive diagnostic ability of naive standardised neuropsychological testing at initial clinical presentation for the differential diagnosis of early stages of bvFTD and AD. For this purpose, we retrospectively evaluated the neuropsychological performance of patients with validated dementia subtype diagnosis based on typical MRI atrophy pattern, CSF dementia biomarker profile and clinical follow-up presentation (Fig. 1). Because researchers in previous studies have described typical cognitive profiles for AD and bvFTD pertaining to single cognitive domains and when using in-depth assessments [11–21], we aimed to investigate typical patterns of impairment in patients with bvFTD in a standard neuropsychological assessment employed in clinical neuropsychological routine.

### Cognitive profile of patients with bvFTD compared with AD

According to current diagnostic criteria, patients with bvFTD present with the core neuropsychological feature of executive dysfunction, whereas episodic memory and other cognitive functions such as visuospatial abilities are either not or only slightly affected [4]. Studies with pathologically confirmed cases have shown high sensitivity (91%) and specificity (83%) for the specified cognitive diagnostic item [5]. However, these studies did not further investigate the specific pattern that patients with bvFTD show on standard neuropsychological test assessments. Previous studies in which investigators have analysed cognitive profiles in more detail have already questioned the current neuropsychological diagnostic item for bvFTD [6, 8, 12–14, 19, 20]. In our sample, patients with clinically documented bvFTD showed impaired performance in a broad range of cognitive domains, including aspects of memory function (*verbal span*, *verbal learning*, *verbal recall*), visuospatial skills, praxis and social cognition to varying degrees. More surprisingly, they also did not show substantial impairment in standard executive function tests when compared with normative sample data or patients with AD. To some extent, patients with AD were in fact significantly more impaired in executive function subdomains. Regarding the diagnostic sensitivity and specificity of these impairments, these results have significant clinical implications for the role of cognitive disturbances in diagnosing bvFTD.

#### Attention and executive function

*Executive function* is an umbrella term of regulatory cognitive processes related to widespread brain regions in frontal as well as parietal areas [27]. Due to frontal atrophy that characterises bvFTD, impaired executive function has consequently been described for this patient group [9, 28, 29]. It has previously been shown that specific executive function assessments can determine parietal and frontal executive functions separately and may thus help to differentiate bvFTD from AD [12, 13]. However, distinct impairment in executive abilities of patients with bvFTD cannot be objectified by using a standard test selection for attention and executive function as we did in the present study. Performance of the patients with bvFTD was heterogeneous across subdomains: they performed similarly to age-matched normative control subjects, except for *phonematic word fluency* and *digit-span backwards*, which is also line with results from other groups [12, 30, 31]. More striking, none of the tested attention or executive subdomains alone was able to discriminate the dementia subtypes or even contribute differentially to their correct classification. In fact, patients with AD reached either similar or even significantly worse scores on executive subdomains compared with patients with bvFTD. Thus, although executive function deficits are among the cognitive impairments of patients with bvFTD, standard neuropsychological tests such as those used in the present study are not sensitive enough to determine differential executive dysfunction in patients with early AD and bvFTD. These results clearly call for a revision of the current diagnostic criteria for bvFTD in which the term *executive function* needs further specification and operationalisation.

#### Memory

Patients with AD performed significantly worse in the subdomains *verbal recall* and *verbal recognition* than patients with bvFTD, and *verbal recall* contributed to classification of AD and bvFTD, as indicated by the discriminant function analysis. However, compared with age-matched control subjects, we found memory dysfunction in one-third to one-half of patients with bvFTD for most memory subdomains. This result is at odds with current diagnostic criteria proposing “relative sparing of episodic memory” in bvFTD and clearly challenges clinical diagnosis at initial presentation.

Our findings are in line with a range of previous studies and growing evidence suggesting that patients with bvFTD may present with significant disturbance of memory function, even in early disease stages [8, 11, 32]. Some studies have indicated that memory deficits in bvFTD may result from dysfunction in prefrontally mediated retrieval control mechanisms and attentional dysfunction [33–35]. More recent data, however, have led to a proposal that neural degeneration in mediotemporal areas such as hippocampal structures may also contribute to amnesia in bvFTD [36, 37]. This latter stance is in line with our finding of one-third of patients with bvFTD showing impaired performance in all aspects of memory capacity, including the subdomain *verbal recognition*, which is particularly sensitive to hippocampal degeneration [38, 39]. Moreover, patients with bvFTD in our sample did not reveal particularly marked executive dysfunction, which could explain the present memory impairment sufficiently.

#### Visuospatial skills

In line with previous findings and current diagnostic criteria, patients with AD showed significantly more impairment in visuospatial skills as assessed by the subdomain *figure copy* than patients with bvFTD. Consequently, this subdomain also contributed to diagnostic classification of dementia subtypes.

#### Language and praxis

Patients with bvFTD showed only slight impairments in naming abilities compared with age-matched normal control subjects, which contrasts with recent findings [40]. A reason for this divergent result might be that patients in that prior study showed much more advanced disease duration (7.2 years on average) than our sample. However, *object naming* was also the least impaired cognitive subdomain for patients with early AD, and thus assessment of language function also did not significantly contribute to the differential diagnosis.

More divergent patterns of cognitive disturbance in our sample were found within the praxis domain: the majority of the patients with AD showed deficits in the subdomains *pantomime of object use* and *imitation of limb gestures.* On the contrary, patients with bvFTD showed major disturbance in the subdomain *imitation of face postures* compared with normative data. These findings validate previously published data, which established disease-specific apraxia profiles for bvFTD and AD [14, 15]. Importantly, both *imitation of face postures* and *imitation of limb postures* differentially contributed to correct group classification within the employed standard test battery, further emphasising the usefulness of testing for apraxia in early disease stages of neurodegenerative diseases, in line with earlier studies [16].

#### Social and emotional cognition

Several independent groups have recently focused on the use of social cognition assessments to differentiate between AD and bvFTD [17, 18]. In our sample, the majority of the bvFTD group but less than half of the AD group was impaired in the subdomain *facial emotion recognition* when compared with normative sample data. For patients with bvFTD, this is in concordance with earlier studies investigating emotion recognition skills in individuals diagnosed with this disease [19–21, 41]. There is evidence that pathological changes in the amygdala, the orbitofrontal cortex and the insula lead to disturbed emotion processing in bvFTD [21]. The ability of patients with AD to recognise facial emotion is discussed more controversially and may also crucially depend on individual disease progression [18, 42].

Surprisingly, despite the clear differences regarding the proportion of impaired cases, we could not find significant group-level differences for the subdomain *facial emotion recognition* at initial clinical presentation. Consequently, the applied social cognition task did not contribute to differentiate between patients with AD and those with bvFTD in our classification analysis. Concerning the domain of social cognition, it has to be taken into account that successful group classification in previous studies also relied on clinical tasks measuring theory of mind abilities and sarcasm detection rather than merely on facial emotion recognition tasks [17, 20, 21]. Nevertheless, our finding of impaired *facial emotion recognition* ability in patients with early bvFTD and proportions of patients with AD clearly warrants further investigation and challenges the perspective of a differential and general impairment of social cognition in bvFTD.

### Limitations

This retrospective clinical study has some methodological and sample-related limitations. Patients with AD were older than those with bvFTD, reflecting disease-specific younger age of onset in bvFTD [1]. Although we statistically verified that age, sex, education, disease duration and MMSE score had no significant impact on the conclusions regarding differences in cognitive performance profiles (see Table 3), this fact may still limit the generalisability of our results. Patients with AD scored slightly lower than patients with bvFTD on the MMSE. The MMSE has previously been reported as being sensitive for measuring core symptoms and thus disease severity in AD but not in bvFTD [43, 44]. Our major aim was to compare patients with dementia of different subtypes in mild to moderate disease stages. Due to the naturalistic setting, disease stage was not evaluated using a single staging instrument developed for both dementia types (e.g., Frontotemporal Lobar Degeneration-Modified Clinical Dementia Rating) [45]. However, we additionally applied the FBI to validate severity of core symptoms in patients with bvFTD, and all our patients showed mild to moderate impairment according to this measure, too. More important, documented disease duration was comparable in both patient groups.

## Conclusions

Patients with bvFTD present with significant impairments in a broad range of cognitive domains at initial clinical presentation. In contrast to current clinical guidelines [4], executive function is neither particularly nor exclusively impaired when assessed using a standard clinical neuropsychological test battery. This result calls for a revision of the neuropsychology item in current diagnostic criteria for bvFTD, including (a) an elaboration and operationalisation of the proposed executive dysfunction and (b) deterioration in other cognitive domains, such as memory, visuoconstruction, praxis and social cognition. We found a significant overlap of bvFTD and AD concerning the profile of cognitive impairments, complicating early differential diagnosis of these dementia subtypes in clinical practice, especially in individual cases. Our results imply that the contribution of a standard neuropsychological assessment to differential diagnosis at initial clinical presentation is limited and argues for the additional use of more specialised and expansive test batteries. Especially, the domains social cognition, attention, praxis and executive function may benefit from more sensitive assessments to display their diagnostic power. Because neuropsychological testing is an economically worthwhile and non-invasive diagnostic procedure, further studies need to be done to investigate specific neuropsychological assessments for differential diagnosis between bvFTD and AD. Whenever such specialised neuropsychological diagnostics cannot be provided, findings of our discriminant analysis indicate that the standard tasks for *verbal recall* and *figure copy* as well as *imitation of face* and *imitation of limb postures* provide a relatively good efficacy for differential diagnosis between bvFTD and AD. Thus, an employed standard test battery should at least cover the domains of memory, visuoconstruction and praxis.

## Abbreviations

AD: Alzheimer’s disease
bvFTD: Behavioural variant of frontotemporal dementia
CERAD: Consortium to Establish a Registry for Alzheimer’s Disease neuropsychological test battery
CFT: Complex Figure Test
CSF: Cerebrospinal fluid
FBI: Frontal Behavioral Inventory
FDG-PET: ^18^F-fluorodeoxyglucose positron emission tomography
MMSE: Mini Mental State Examination
MRI: Magnetic resonance imaging
RWT: Regensburg Word Fluency Test
SEA: Social Cognition and Emotional Assessment
TMT: Trail Making Test, VLMT, Verbal Learning and Memory Test
